# Supplementary material for: Are carbon emissions trading and green financial instruments synergistic? -Comprehensive quantitative research based on content analysis
Source: PLoS One. 2024 Mar 7;19(3):e0298601. doi: 10.1371/journal.pone.0298601 (PMC10919601; doi:10.1371/journal.pone.0298601)
Supplement: S1 Data — (ZIP) [file pone.0298601.s001.zip › Data/地级市Z_F工作报告.zip!/╕ú╜¿/╒─╓▌╩╨/└┤╫╘╣1⁄2╓┌║┼í╛┬φ┐╦╩2╛▌═°í┐_2017.docx]

各位代表：

现在，我代表漳州市人民政府，向大会报告政府工作，请予审议，并请市政协委员和其他列席人员提出意见。

一、2016年和过去五年工作回顾

过去一年，在以习近平同志为核心的党中央坚强领导下，我市各级政府深入贯彻习总书记系列重要讲话精神和对福建工作的重要指示，认真贯彻落实中央、省和市委的各项决策部署，坚持稳中求进工作总基调，坚持新发展理念，坚持以推进供给侧结构性改革为主线，扎实开展“三个建设年和一大攻坚战”及“冲刺100天，完成全年目标任务”活动，紧盯目标不动摇、一二三产齐发力，经济社会发展取得新成效。

全市生产总值3125.34亿元，增长9.3%；一般公共预算总收入287.68亿元，增长4.7%，地方一般公共预算收入187.64亿元，增长4.8%；规模工业增加值1396.33亿元，增长8.1%；固定资产投资2827.93亿元，增长12.4%；外贸出口72.68亿美元，增长3.3%；实际利用外资11.64亿美元，增长7.2%；社会消费品零售总额875.59亿元，增长12.7%；居民消费价格总水平上涨1.5%；城镇居民人均可支配收入30726元，增长9.4%；农民人均可支配收入15320元，增长10.5%；城镇登记失业率2.24%；人口自然增长率10.8‰。年度节能减排降碳任务全面完成。主要工作和成效是：

（一）追问题解难题，经济保持平稳增长。坚守“三条底线”，深化“追问题、解难题、促发展”和“政策不睡觉、落地见成效”活动，出台工业经济稳定增长、现代服务业发展、外贸出口增长等政策措施，设立市级产业股权投资引导基金，累计发放扶持资金2.85亿元，减免涉企税费30.8亿元，使用“过桥”资金37.2亿元。注重“五个一批”项目滚动接续，推动项目加快落地，514个市级在建重点项目完成投资1480亿元，98个省级在建重点项目完成投资401亿元，121个列入省行动计划在建项目完成投资422亿元，均超额完成年度投资任务；组织实施两批次21个补短板工程包183个项目，完成投资197.7亿元；争取国家专项建设基金29.7亿元；6个PPP项目列入财政部第三批PPP示范性项目；新开工亿元以上项目250个、建成或部分建成135个。福建古雷石化公司正式成立，古雷整岛搬迁和征地清海工作全面完成，锦江石化芳烃一体化项目正式签约，双鱼岛填海工程竣工验收，漳州核电一期工程“两评”报告获批。设立中小企业贷款风险补偿基金，金融机构各项贷款余额增长13.7%；列入省级重点上市后备企业51家，新增新三板挂牌12家、海交中心挂牌10家；企业直接融资260.1亿元、增长11.7%。县域经济实力得到增强，长泰县被评为全省县域经济实力“十强”县，4个县被评为县域经济发展“十佳”县。

（二）强化供需对接，供给侧改革有效推进。出台“三去一降一补”具体措施，实施产业龙头促进计划、新兴产业倍增计划和创新示范工程，新增规模工业企业120家、高新技术企业21家、国家级企业技术中心2个、省级企业工程技术研究中心3个、专利授权3500件；成立汽车、钢铁产业联盟。服务业加快发展，第三产业增加值增长13%，增幅位居全省首位；入选中国“电子商务百佳城市”，电子商务交易额230亿元，增长65%；东山列入首批国家全域旅游示范区创建单位，南靖土楼“四菜一汤”被外交部列为国家对外交流名片，全市接待游客2640万人次、增长18.3%，旅游总收入300亿元、增长25%；商品住房销售面积增长40%。国家级现代农业示范区建设步伐加快，新增设施农业2.2万亩、“三品一标”87个，建设高标准农田13.8万亩、补充耕地1.13万亩，134家农业企业获批第八轮农业产业化省级重点龙头企业，总数位居全省首位。

（三）突出试点试验，改革开放活力释放。年度各项改革任务如期完成。构建开放型经济新体制综合试点试验工作取得实效，年度13个重点推进项目进展顺利。建立并公布“八张清单”，在全省率先实现乡镇权责清单全覆盖；组建高新区“商务110”，推行“模拟审批”“一口受理、一网通办”，推进“会审制”试点，在全国率先开展“多评一表、多图一审”试点改革；深化“五证合一、一照一码”改革，营改增试点全面推开，推行国地税联合办税，营商环境进一步优化。国企改革稳步推进，国有资产进一步保值增值。深化漳台合作，漳台贸易额15.7亿元、增长10%，实际利用台资7.3亿美元，“农博会·花博会”成功举办。开展“漳州味·世界行”系列展销活动，与东盟贸易额11.6亿美元。

（四）着力同城发展，城乡面貌明显改观。推进“多规合一”试点，完成《富美漳州战略规划》编制。开展市政提升“五千工程”，更新改造供水管网144公里、城市道路155公里，新增绿道133公里、公园绿地2937亩、公共停车位3643个、公交车168辆。“达达通”公共自行车项目建成投用。市域一体化和厦漳同城化步伐加快，14个国省干线“互联互通”项目加快建设，21个厦漳同城化重大项目进展顺利。中心城区环境整治力度加大，通过全国文明城市年度测评和国家卫生城市复审。闽南文化生态产业走廊示范段获“全国生态文化示范基地”称号，西环城路景观整治提升暨绿道建设工程荣获“2016年中国人居环境范例奖”。实施流动人口居住证制度，长泰县列入第三批国家新型城镇化综合试点，角美镇成为全国深化城镇基础设施投融资模式创新特色镇。实施新一轮“千村整治、百村示范”美丽乡村建设工程，全国美丽乡村标准化试点会议在长泰召开，3个村入选全国美丽宜居村庄，10个村入选中国传统村落。

（五）深化环境治理，生态优势更加凸显。扎实推进生态文明先行示范区建设，深入实施“七个五”生态建设行动计划，启动九龙江流域生态水系先行示范工程。市区内河、“五小”污染企业、养殖业污染整治扎实推进，九龙江流域（漳州段）Ⅲ类水质达标率93.8%。开展扬尘专项整治，淘汰黄标车10491辆，全市空气质量优良率平均97.8%。新改扩建污水处理厂4座，新建污水配套管网128公里。规范推进排污权交易，191家企业完成排污权核定。造林绿化15.4万亩，治理水土流失28万亩。荣获“中国十佳绿色生态旅游城市”“全国绿化模范城市”称号，新增3个“国家生态县（区）”，4个县（区）通过国家生态县（区）考核验收。

（六）推动成果共享，社会事业全面进步。21件为民办实事项目基本完成。全力打好脱贫攻坚战，38486人、54个贫困村实现脱贫，造福工程搬迁39674人，完成4132户贫困危房户危房修缮，解决989户贫困无房户住房问题。城镇新增就业5.14万人，各群体养老金水平稳步提高，城乡居民医保财政补助继续增加，保障性安居工程建设超额完成任务。 新增中小学、幼儿园学位14821个，提前一年全部通过国家义务教育发展基本均衡县（区）验收。新建续建卫生项目83个，新增病床位1625张、卫技人员1450名， 建成基层医疗机构中医馆23家；医改工作有序推进，医疗费用增速和药占比下降，医保管理职责整合到位。计生工作、二孩政策有效落实。实现全市城乡居民大病保险全覆盖，列入第二批国家级医养结合试点。社会福利中心动工建设。实施文艺精品创作工程，一批优秀剧目荣获国家级奖项。全民健身广泛开展，妇女儿童工作得到加强，人防、地方志、民族宗教等工作和残疾人、慈善事业发展取得新进步。安全生产标准化建设“三年提升工程”全面完成，道路交通安全综合整治三年提升工程全面启动，食品药品安全、寄递物流等新业态和网络安全监管力度加大。“五馆一歌”加快建设。“平安漳州”建设扎实推进，“六五”普法顺利通过省级验收，“七五”普法规划制定实施，“漳州110”勤务改革正式启动，群众安全感满意度保持在95%以上。军政军民团结巩固发展,实现“全国双拥模范城”六连冠。漳州数字档案馆成为全省首家“全国示范数字档案馆”。苏区老区、对口支援、山海协作、援藏援疆援宁等工作有效开展。

（七）践行“两学一做”，自身建设得到加强。深入开展“两学一做”学习教育，认真贯彻中央八项规定精神，持续反对“四风”，强化“马上就办”，大力整治“庸懒散拖”，机关作风明显改善。市本级“三公”经费支出下降15.9%。公车改革顺利推进。认真执行人大决定决议，自觉接受人大监督、政协监督和社会监督，办理人大代表建议165件、政协委员提案296件，办复率100%，政府与政协、法院、工会互动联系机制不断完善，各民主党派参政议政、民主监督富有成效。政务公开和权力运行网上公开全面推进，行政监察、审计监督继续加强，党风廉政建设和反腐败工作取得实效。

各位代表：

2016年工作任务的完成，标志着本届政府工作顺利收官。

五年来，我们精准施策稳增长，综合实力得到新提升。地区生产总值年均增长11.1%，增幅近三年连续蝉联全省第一。一般公共预算总收入、地方公共预算收入提前实现比2010年翻一番以上。荣获第四届全国文明城市、国家森林城市、中国温泉之城、全国科技进步先进市等称号。2014、2015年省对市绩效考评总分分别位居全省第一、第二。

五年来，我们一二三产齐发力，转型升级取得新实效。强化工业主导地位，累计完成工业投资4360亿元，技改投资2345.8亿元，年均增长37%，新增规模工业企业669家，2016年规模工业增加值是2011年的1.9倍，食品、装备制造两大产业产值突破千亿元。现代服务业加快发展，成为经济增长新动能。现代农业巩固提速，全市域获批国家级现代农业示范区。创新驱动战略深入实施，高新技术产业主营业务收入、全社会研发投入分别是2011年的2.5倍和1.9倍。

五年来，我们注重统筹促协调，城乡环境呈现新变化。坚持“田园都市、生态之城”定位，全面推进新型城镇化，中心城区规划面积从250平方公里扩大到686平方公里，建设用地由85.1平方公里增加到108.1平方公里，城镇化率五年提高8.1个百分点。全面推进绿色发展，五年来万元GDP能耗下降26.6%。

五年来，我们持续投入补短板，民生福祉实现新改善。新增财力向民生领域倾斜，民生支出占财政支出77.1%，城乡居民人均可支配收入年均分别增长11.4%和12.3%，累计新增城镇就业26.7万人，转移农村劳动力36万人，8.43万人实现脱贫。城乡居民社会养老保险制度实现一体化，基本医疗保险制度实现全覆盖，每千人口病床位数和卫技人员数分别从2.49张、2.84名提高到4.42张、4.85名。公共文化服务体系更加健全。

各位代表，成绩的取得来之不易，得益于省委、省政府和市委的正确领导，得益于全市人民齐心协力、团结奋斗和各方面的大力支持。在此，我代表市人民政府，向全市人民，向人大代表、政协委员、各民主党派、工商联、各人民团体和社会各界人士，向中央、省驻漳单位和驻漳部队、武警官兵、公安民警，向关心支持漳州发展的台港澳同胞、海外乡亲和国际友人，表示衷心的感谢！

在看到成绩的同时，我们也清醒地认识到发展中还存在不少困难和问题，主要是：经济实力不强，人均水平偏低，产业结构不优，新兴产业和新动能还比较弱小；投资增速回落，缺少支撑经济转型发展的大项目好项目；创新能力不足，高层次人才紧缺，科技研发和转化能力偏弱；城乡区域发展不均衡，城镇化率偏低，城乡治理和规划建设水平有待提高；脱贫攻坚任务仍然艰巨；生态环境保护压力较大，应对重大自然灾害能力有待提升；基础设施欠账较多，优质教育、医疗资源不足，基本公共服务存在不少短板；体制机制创新和政府职能转变还不到位，不敢担当、慢作为、中梗阻和不贪不占也不为的问题仍不同程度存在，发展环境仍需优化等等。我们一定要高度重视这些问题，采取更加有力措施认真加以解决。

二、谋划好未来五年工作，全力加快建设富美新漳州

按照省第十次党代会和市第十一次党代会的部署，今后五年市政府工作的总体要求是：深入贯彻以习近平同志为核心的党中央治国理政新理念新思想新战略，紧紧围绕“五位一体”总体布局和“四个全面”战略布局，全面落实新的发展理念，紧扣“再上新台阶、建设新福建”的中心任务，坚持稳中求进工作总基调，以提高经济发展质量和效益为中心，以推进供给侧结构性改革为主线，持续“田园都市、生态之城”发展定位，实现经济总量、发展质量、人均均量、宜居环境、人民群众获得感“五个提升”，确保全面小康社会如期建成、富美新漳州建设再上新台阶。

今后五年，我们的主要奋斗目标是：

（一）建设产业升级的实力漳州。深化供给侧结构性改革，落实“三去一降一补”，扩大有效供给和高端供给，地区生产总值年均增长10%左右，规模工业总产值突破万亿元。坚持工业强市，实施工业跨越发展行动计划和“漳州制造2025”计划，大力发展智能制造、绿色制造，产业迈向中高端，四大主导产业产值突破7000亿元，四大战略性新兴产业产值达1500亿元。坚持服务业兴市，着力金融加强、物流加速、电商做大、房地产去库存、旅游做人气、健康养生做品质，促进现代服务业扩总量优供给。坚持农业稳市，发挥“一区两园”示范带动作用，围绕品种、品质、品牌，促进农业接二连三、融合发展。

（二）建设开放创新的活力漳州。坚持创新驱动发展战略，推进科技创新、制度创新、模式创新，强化企业创新主体作用，科技进步贡献率提高到58%以上，努力建设创新型城市。突破重点领域和关键环节改革，构建开放型经济新体制，形成一批可复制、可推广的经验和模式。加快建设深化两岸产业合作示范区和古雷深化两岸石化产业合作示范区，发挥“海丝”重要节点城市优势，推进厦漳泉大都市区同城化，拓宽闽粤经贸合作平台，拓展对内对外开放新空间。

（三）建设富美宜居的幸福漳州。强化脱贫攻坚，确保2018年现行标准下贫困人口全部脱贫，贫困县全部摘帽，全面建成小康社会。坚持“田园都市、生态之城”城市定位，大力实施“中心东移、跨江南扩、面海拓展”发展战略，做大做强做美中心城市，充分彰显“水城、绿城、花城、历史文化名城”特色。全面推进生态水系建设，率先建成国家生态文明示范区，加快建设美丽漳州。持续扩大优质教育、医疗等公共服务供给，健全完善公共服务体系，推动民生福祉获得较大提升。

三、扎实做好2017年工作

新的一年，我们要按照市委要求，紧紧围绕“再上新台阶、建设新福建”的中心任务，坚持稳中求进工作总基调，以提高经济发展质量和效益为中心，以推进供给侧结构性改革为主线，持续深化“三个建设年和一大攻坚战”活动，突出“三抓三比”，开展“十项竞赛”，全力促进经济平稳健康发展和社会和谐稳定，实现富美新漳州建设良好开局，以优异的成绩迎接党的十九大胜利召开。

经济社会发展主要预期目标是：全市生产总值增长9.5%以上；一般公共预算总收入增长5%，地方一般公共预算收入增长4%；固定资产投资增长16%；规模工业增加值增长8.5%以上，规模工业总产值增长8.8%以上；外贸出口增长3%；实际利用外资增长5%；社会消费品零售总额增长12%，居民消费价格总水平涨幅控制在3%左右；城镇登记失业率控制在3.6%以内；城镇居民和农民人均可支配收入分别增长10%；落实节能减排降碳任务。

实现上述目标，必须着重做好以下七个方面的工作：

（一）发力供给侧，着力经济平稳增长

扩大有效需求优供给。发挥投资、消费和出口的拉动作用，落实“去降补”年度任务，开展“增品种、提品质、创品牌”专项行动。健全“五个一批”项目管理机制，按照“两个50%以上”和每月开工一批亿元以上项目要求，安排重点项目548个，年度投资1248亿元，新开工市级重点项目200个，建成或部分建成148个，全力推动漳州核电、金龙客车、中海油LNG等重大项目建设，力促猛狮新能源、英博雪津三期等项目建成投产或部分投产。继续实施补短板工程包，争取更多国家专项建设基金。规范推广PPP模式，建立健全吸引社会资本的体制机制，进一步激活民间投资。实施“促升级、扩消费十大行动”，丰富消费业态，拓展消费热点。落实稳出口政策措施，开展出口企业“同线同标同质”活动，拓展“漳州味?世界行”成效。

优化投资环境降成本。深化追问题、解难题、促发展，落实并出台一批降成本政策，为实体经济压减税费、融资、物流、用能、技改等生产运营成本，提高企业市场竞争力。降低各类交易成本特别是制度性交易成本，落实前期代办制，继续推行模拟审批、并联审批，推广一网通办、多评一表、多图一审等服务，提高审批效率。推动企业降本增效，鼓励企业通过技改、兼并重组、上市等方式做大做强，出清过剩产能。

突出创新引领强动力。实施“科技小巨人”成长行动计划，力争新增高新技术企业20家、省级创新型企业5家、省级科技型企业60家，加快培育创新型企业集群。抓好一批重大创新平台项目，建设一批高水平的创客空间、创新创业中心和新兴产业“双创”示范基地，推动华为云计算数据中心、智能汽车小镇、北大科技医疗小镇、中欧食品谷等项目落地建设，争取新增省级企业工程技术研究中心5家、省级重点实验室2家。建立以“用”为导向的产学研结合机制，实施科技创新链与产业链精准对接工程，鼓励龙头企业组建产学研用创新联盟，推动更多科技成果快速产业化。建立更加灵活务实的人才引育激励机制，优化教育、医疗配套服务，建设人才公寓，更加广泛汇聚创新资源。

（二）转型促发展，着力壮大实体经济

强化重点区域支撑。继续举全市之力支持古雷建设，全面提升规划布局与安全环保水平，加快推进填海造地等重大基础工程，加快建设中石化古雷炼化一体化、锦江石化芳烃一体化等重大项目，力促台湾中石化烯烃芳烃一体化、延长石化炼化一体化等重大在谈项目早日落地，推动腾龙翔鹭资产重组，争取PX、PTA早日恢复试生产，推动国际油品交易中心建设，加速壮大石化产业。厦门港南岸新城要全面对接厦门，抓好福欣特钢二期、惠尔康生物科技产业、招商局?芯云谷、双鱼岛等项目建设，支持龙海加快南太武南溪湾组团规划建设，加快提升城市形态、区域价值、产业层次。漳州高新区要注重完善功能配套，推进“退城入园”、产城融合，加快建设智能制造产业示范园、健康食品产业示范园和中盟科技产业园等平台项目。环东山岛经济区域重点加快海洋生物产业园、东山玻璃及新材料产业园、乌山景区综合开发等项目。开展工业园区建设竞赛，实施工业园区提升工程，加强园区基础设施建设，种好招商引资的“梧桐树”。

推动工业跨越提升。开展创新转型项目竞赛，突出“4+4”产业，强化龙头带动引领，促进产业链上下延伸，加速产业集群集聚，做大做优工业增量。培育壮大省市级龙头企业50家、高成长型企业120家，新增（新上）规模工业企业120家。积极培育新兴产业生力军，筛选一批高成长企业、新增长点项目，实行全成长过程扶持、全要素资源支持，加快培育“专精特新”中小企业。实施新一轮技术改造工程，引导企业加大技改投入，完成技改项目投资680亿元以上。强化质量管理，实施工业产品、消费品和出口商品三大质量提升工程，筛选一批质量、品牌示范企业，推行个性化定制、标准化生产、品牌化运营，让好东西卖出好价钱。深化“两化”融合，推广数字车间、智能装备、网络协同制造。引导重点行业成立产业联盟，促进企业互为配套、互为用户。用足港口优势，做大做强海洋经济。

促进服务业加快发展。开展现代服务业项目竞赛，像抓项目、抓工业一样抓服务业，加大规划引领和项目策划推进力度。金融业注重增实力、强服务、化风险，深化“引银入漳”，发展绿色金融和普惠金融，健全政策性融资担保体系，支持企业设立财务公司；鼓励产业基金、融资租赁、股权交易入漳搭建平台，探索成立产业投资公司，促进金融资本与产业资本有效对接、深度融合。物流业注重提质、降本、增效，加快发展智慧物流、冷链物流和第三方、第四方物流，加快建设5个综合物流园区和若干个专业物流中心、配送中心，推进交通物流融合发展和“互联网+物流”发展；扶持发展跨境电商、农村电商，加大企业电商化改造，培育一批国家级、省级电子商务示范园区和企业。旅游业注重文化创意、景点提升、配套服务，充分挖掘我市深厚历史文化内涵，加快策划一批旅游文化项目，推动漳州古城文化旅游、龙海白塘湾国际旅游、东山生态旅游岛综合开发、常山天窗坪仙境山庄等项目建设，促进旅游与文化深度融合，打响“清新福建?花样漳州”品牌，打造生态文化旅游目的地。房地产注重去库存、优供给、稳市场，加快解决房屋“两证”遗留问题，促进房地产市场健康发展。健康服务业注重降门槛、提品质、扩供给，支持多元市场创办养老服务机构，争取每个县（市、区）都引进1家以上居家养老服务组织，打造若干特色健康养老服务示范区，扩大社区健康养老服务供给。

（三）特色创优势，着力提升农业质效

拓展提升特色农业。围绕深化农业供给侧结构性改革，开展特色现代农业项目竞赛，不盲目追求面积产量增长，大力发展品牌农业、智慧农业、生态农业，不断增加绿色优质农产品供给。稳定粮食生产，确保粮食播种面积169万亩、产量68万吨。做优九大特色现代农业，做大“三品一标”，新增“三品一标”60个。实施农产品加工提升工程，培育一批产值上10亿元农产品精深加工领军企业。全面推广设施农业，提升“一区两园”、24个特色产业“核心示范园”水平，加快建设一批示范基地和窗口项目，新增设施农业2万亩。支持发展现代海洋渔业。实施“菜篮子”增品种、提品质工程，保障市场有效供给。积极推进“互联网+特色现代农业”，培育发展农业新业态，提高农业综合效益，努力让农业强起来、农民富起来、农村美起来。

推进农业可持续发展。启动国家农业可持续发展试验示范区建设。实施农产品质量安全“1213”行动计划，倡导农业绿色生产，狠抓农产品质量安全监管，切实管住舌尖上的安全。实施“四个专项行动”，推广绿色有机肥、减量清洁生产技术和生态环保养殖模式，完善农药化肥和农产品质量安全追溯体系。加强动植物防疫。保护好永久基本农田，实施“地力提升1112工程”，新建高标准农田11.1万亩。加强以水利为重点的农村基础设施建设，抓好小型农田水利重点县建设和灌区节水改造。强化基层农技推广体系建设。做好第三次全国农业普查工作。

创新农业经营体系。完成农村土地承包经营权确权登记颁证工作，落实农村土地“三权分置”办法，完善土地流转服务体系，发展多种形式适度规模经营。深化国有农场改革。开展示范性家庭农场创建活动，推进农民合作社规范化，培育新型职业农民和社会化服务组织。全面启动实施国有林场改革。创新农业投入机制和财政、金融支农方式，提升“三农”综合保险水平。

（四）构建新体制，着力增创竞争优势

强化改革试点带动。聚焦《构建开放型经济新体制综合试点试验工作方案》的五大重点任务，落实好4项改革措施和39个首批重点推进项目。深化行政审批制度改革，进一步精简审批事项，推进经济社会管理权限下放特殊区域。试行市场准入负面清单制度，健全“双随机一公开”监管机制，建设统一的政务服务平台和投资审批监管平台。深化商事制度改革，加快推进县（市、区）、市属开发区相对集中行政许可权改革。深化行政体制改革，加快推进事业单位分类改革，重点推进综合行政执法体制改革，统筹推进重点区域、经济发达镇以及城市管理、教育卫生、生态环保、司法体制等领域体制改革。深化财税体制改革，更加重视做好财政挖潜增收工作。建立健全政府购买服务制度体系。持续推进国企改革，加快政府融资平台市场化转型，促进国企做大做强。

推动漳台深度融合。创新“两岸一家亲”融合机制，深化漳台产业对接升级计划，密切与台湾工商团体、行业协会联系，推进石化、集成电路、光电、精密机械、新材料等产业深度对接，推动产业合作向研发创新、打造品牌、制定标准迈进。加快古雷石化基地开发建设，打造深化两岸石化产业合作示范区。积极引进台资银行、保险、证券等金融机构。推进漳浦台湾农民创业园建设，提升漳台农业合作水平。支持在漳台资企业转型升级，维护台胞台商合法权益。办好第九届海峡论坛相关活动。推动漳台乡镇、同名村、社区“点对点”共建，深化漳台姓氏宗亲、民间信仰、民俗文化交流。

扩大对外开放格局。健全招商协调推进机制，组建商机对接中心，推广高新区“商务110”平台，加强招商督查考评。加大五类企业招商力度，引进填补产业链缺失项目。开展“漳商回乡行”活动，促进优势资源回归。加快台商投资区保税物流中心（B型）建设，优化口岸通关环境。注重“走出去”战略实效，加强“一带一路”沿线国家和地区的双向投资与贸易合作。传承月港辉煌，加强“海丝”遗址保护，打造“海丝”文化品牌。紧密联系更多港澳侨漳籍乡亲，加强与新生代乡亲交往。

（五）加快城镇化，着力城乡一体发展

做大做美中心城区。落实规划委员会制度，实施规划提升工程，科学划定城市开发边界，推进“多规合一”试点，扩大城市设计覆盖面。开展新一批市政线网“六千工程”，开工建设芝山南路跨江大桥、金峰大桥等，加快南江滨路、联十一线漳州段等市政道路建设，抓好智能电网、地下综合管廊建设，全年新改扩建城区雨水管网、污水管网、供水管网、城市道路各120公里以上，城市绿道130公里以上、燃气管网80公里以上；实施公共交通提升工程，新增小型公交50辆，新建“达达通”公共自行车网点100个、新增自行车3000辆，续建新建公共停车设施32个，新增车位2500个以上。全面开展中心城区生态环境提升专项行动，加快建设“五湖四海”，延伸拓展“闽南水乡”，打通内河水系，建设海绵城市。加大旧城改造力度，统筹城市旧区存量用地，降低城市密度，加快城市修补和生态修复。

深化宜居城乡建设。巩固全国文明城市创建成果，加强“门前三包”执法，关停31个市区垃圾中转站，推行垃圾直运机制，推动垃圾不乱丢、黄土不露天、车辆不违章、经营不占道、广告不乱贴。持续开展占用地上地下公共空间专项整治，加快改造无物业小区。推进新一批城区主干道“白改黑”和立面改造工程，加强县域交界处、城乡结合部和“三边三节点”环境整治。深化“两违”治理，完成拆违面积600万平方米以上。扎实推进“千村整治、百村示范”美丽乡村建设工程，整治100个以上村庄，创建12个美丽乡村示范村。加强古村落、特色景观村落、历史文化名镇名村保护传承，鼓励发展特色民宿和“农家乐”。

推进区域协同发展。主动推进厦漳同城化，加快厦漳同城大道、福厦（漳）高铁客运专线、厦蓉高速扩容工程等项目建设，力促厦门地铁6号线漳州（角美）段、厦漳城际轨道R3线开工建设。加快市内同城化步伐，推进14个国省干线“互联互通”项目和4个高速公路项目建设，着力打通内陆山区出海通道。加快闽粤经济合作区建设，推动两地合作取得突破。

加快建设特色小城市（城镇）。加强县城规划建设，加快推进一批省级小城市培育试点、小城镇综合改革建设试点以及特色小镇创建工作。开展特色小镇项目竞赛，支持各地结合美丽乡村、古村落保护、产业园区建设等，培育一批产业小镇、旅游小镇、文化小镇。加速中心城区全域城市化，有序推进镇改街、村改居，推动居住证制度全覆盖，稳步推进城镇基本公共服务覆盖全部常住人口。

（六）创建示范区，着力保护生态环境

提升生态创建水平。深入实施“七个五”生态建设行动计划，全面推进国家生态文明示范区创建工作。加大红树林、基本农田、矿产资源等保护，推进矿山生态环境恢复治理，补充耕地9500亩，完成造林绿化10.25万亩、森林抚育13万亩、封山育林8.31万亩、水土流失综合治理28万亩。更加重视海洋生态环境保护，强化自然岸线管控，开展盗采海砂、海水养殖污染整治。全面落实河长责任制和生态补偿机制，加强环境执法，推行环境监管网格化管理。

加大“三铁”治污力度。市县乡村四级联动，“气质、水质、土质”一起抓，让漳州的天更蓝、地更绿、水更清。强化大气污染防治，抓好四个专项整治，清理中心城区小烟囱，基本淘汰黄标车，改善空气质量。开展生态水系项目竞赛，加快实施九龙江流域生态水系先行示范工程和万里安全生态水系建设，综合治理“一库两溪三河”流域水系，加快城市内河黑臭水体治理，推进农村垃圾、污水治理及农业面源污染整治，完成流域范围“五小”企业污染整治，全面提升水体质量。制定饮用水源库区保护规划，强化饮用水源安全保障。开展土壤污染状况详查，启动土壤污染治理与修复试点示范工程，加强重金属、固体废弃物、持久性有机污染物治理。

狠抓节能减排攻坚。严格落实节能减排责任制和环保监管“一岗双责”，严把新上项目环保准入门槛。加大工业污染减排力度，淘汰落后产能设备，推动燃煤电厂“超低排放”改造和燃煤锅炉（炉窑）煤改工程，促进造纸行业整合提升。加快城镇污水厂和配套管网建设，加强城中村污水的截流收集和处理，提升污水、垃圾处理率。发展循环经济和绿色清洁生产，推动工业园区集中供热、制冷。

（七）增强幸福感，着力发展民生事业

全力打好精准脱贫攻坚战。扎实推进脱贫攻坚战三年行动，深入实施“七大脱贫攻坚重点工程”，开展精准脱贫攻坚竞赛，确保完成脱贫22816人、79个村。建设省市集中安置区（点）10个以上，完成造福工程搬迁2000户6000人以上。加强贫困地区基础设施和民生工程建设，实现贫困村“七通”。推进产业扶贫项目落地，每个贫困村培育一个以上壮大集体经济收入项目。加快建立完善稳定脱贫长效机制。探索开展可持续的商业保险，为因病致贫的贫困户增加保障。

提升社会保障水平。实施20项为民办实事项目。拓宽就业渠道，全年新增城镇就业4.4万人。完善征迁补偿机制，帮助引导失地、失海农民二次就业创业。推进“三保合一”，实施全民参保任务计划。推动农村低保制度与扶贫开发政策有效衔接，农村低保标准提高500元以上，实现与扶贫标准“两线合一”，落实特困人员救助供养新政策。加快保障性安居工程建设和棚户区改造，新建保障性安居工程1.4万套。大力发展残疾人事业、社会福利和慈善事业，保障妇女儿童、青少年、老年人合法权益，关爱农村留守儿童的健康成长。

扩大健康服务供给。实施“名医、名科、名院”工程，加快建设一批高等级医院，加强产科、儿科等薄弱学科建设，新增床位1000张、卫技人员1000名。深化医药卫生体制改革，推动医管委实质性运作、市医保局有序运转，抓好公立医院综合改革、药品和医用耗材联合限价采购。深化基层医药卫生体制综合改革，推动乡村卫生服务一体化管理，开展慢性病分级诊疗和家庭医生签约服务试点。扶持中医药产业发展。加大精神卫生机构建设。扶持发展“医养结合”，抓好省级四种类型医养结合试点工作。继续抓好计划生育工作，促进人口均衡发展。

办好教育文化事业。扩大普惠性学前教育资源，全市新建17所公办幼儿园。加快优质高中建设发展和薄弱高中改造提升，抓好农村薄弱学校改造、城区中小学校扩容，推进“义务教育管理标准化学校”建设。扶持高校发展，加快建设市职教园区。实施免费特殊教育，重视发展终身教育和老年教育。加强教师队伍建设。推动民办教育健康有序发展。挖掘地域文化特色，培育壮大文化创意产业。实施优秀传统文化传承工程和红色文化工程，重视文物和非遗保护，做好“五古丰登”项目修缮，支持东溪窑、南胜窑等遗址申报世界文化遗产，推动“五馆一歌”建成投用。推进闽南文化生态保护示范区建设。广泛开展全民健身活动，办好第十三届市运会、第十届老年人体育健身大会。做好《漳州文库》编撰、漳州市志续志工作。

加强和创新社会治理。深入开展“七五”普法活动，加强和改进信访调解工作，有效预防和化解矛盾纠纷。深化“平安漳州”建设，完善立体化社会治安防控体系，强化“五大领域”风险预警防控。严格落实安全生产“党政同责、一岗双责、失职追责”和“三个必须”，推行企业安全生产标准化建设。开展道路交通安全综合整治三年提升工程，整治城区“十大”交通乱象。开展食品安全放心市创建活动，深化“餐桌污染”治理，加强食品药品安全监管。完善防灾减灾体系，提高自然灾害防御能力。支持工会、共青团、妇联、科协、残联等人民团体发挥更大作用。加强民族工作，依法管理宗教事务，强化司法行政、国防教育、国防动员、民兵预备役、海防、支前双拥、打私、人防工作。

四、推进服务型政府建设

建设富美新漳州，顺应人民新期待，政府要有新状态、新作为。要牢固树立“四个意识”特别是核心意识、看齐意识，坚决维护以习近平同志为核心的党中央权威，不折不扣把中央、省和市委的决策部署贯彻落实到位。

强化责任担当。以新理念引领新实践，以新担当创造新业绩，破除思维定势和体制障碍，开拓漳州发展新境界。脚踏实地讲认真，增强政府执行力，紧盯目标、挂图作战，量化实化政府工作，以“钉钉子”精神狠抓工作落实。制定“正向激励政策包”，建立容错纠错机制。始终坚持以人民为中心，多听民声、多掌民情、多谋民事、多解民忧、多顺民意，人民群众期盼的，尽量多做；对人民群众有利的，尽力做好，真正让群众在发展中感受到更多的“获得感”。

推进依法行政。坚持法无授权不可为、法定职责必须为，严格遵守宪法和法律，学法懂法、守法用法，依法决策、依法办事，在党的领导下、在法治轨道上开展工作。衔接配合市人大做好立法工作，依法制定规章、规范性文件，建立健全法律顾问制度。自觉接受人大及其常委会监督、政协民主监督、社会监督和舆论监督，加强行政监察和审计监督。推进政务“五公开”，让权力在阳光下运行。

优化营商环境。持续减权限权放权，开展“简政便民”专项行动，全面清理各种证明和手续，让企业和群众办事更省时、省力、省钱、省心。全面推进“互联网+政务服务”，文件多走网路，百姓少跑马路。重承诺、讲规则，等距离交往，无差别服务，守住“亲”“清”底线，构建政商关系新生态，营造好的政策环境、市场环境、服务环境、社会环境、生态环境，让企业守法经营、安心发展。

坚持严政廉政。“把自己的手洗干净，把自己的腰杆挺直”。坚定不移推进全面从严治党，履行主体责任，突出“五抓五看”，把党风廉政建设落实到位。认真践行“三严三实”，严格执行《准则》《条例》，严明政治纪律、政治规矩，严格党内监督，严肃党内政治生活，持之以恒落实中央八项规定精神。加强招投标、工程建设、土地出让、政府采购等重点领域和环节的监管，用制度管人管事管钱。

各位代表，新时期，新使命，新目标，新期待。让我们紧密团结在以习近平同志为核心的党中央周围，在省委、省政府和市委的正确领导下，解放思想、开拓进取，凝心聚力、奋力拼搏，努力在新的起点上加快建设富美新漳州，为“再上新台阶、建设新福建”，为全面建成小康社会，作出新的更大贡献！
